# Cold Bed Chambers

**Published:** 1873-10

**Authors:** 


### COLD BED CHAMBERS

always imperil health and invite fatal diseases. Robust persons may safely sleep in a temperature of forty or under, but the old, the infant and the frail, should never sleep in a room where the atmosphere is much under fifty degrees Fahrenheit.

All know the danger of going direct into the cold from a very warm room. Very few rooms, churches, theatres and the like, are ever warmer than seventy degrees. If it is freezing out of doors it is thirty degrees—the difference being forty degrees more. Persons will be chilled by such a change in ten minutes, although they may be actively walking.

But to lie still in bed, nothing to promote the circulation, and breathe for hours an atmosphere of forty and even fifty degrees, when the lungs are always at ninety-eight, is too great a change. Many persons wake up in the morning with inflammation of the lungs who went to bed well, and are surprised that this should be the case. The cause may often be found in sleeping in a room the window of which had been foolishly hoisted for ventilation. The water cure journals of the country have done an incalculable injury by the blind and indiscriminate advice of hoisting the window at night.

The rule should be, every where, during the part of the year when fires are kept burning, to avoid hoisting outside windows. It is safer and better to leave the chamber door open, as also the fireplace—then there is a draft up the chimney, while the room is not so likely to become cold. If there is some fire in the room all night the window may be opened an inch. It is safer to sleep in a bad air all night with a temperature over fifty, than in a pure air with a temperature under forty. The bad air may sicken you but cannot kill you; the cold air can and does kill very often.

